# Potential effects of environmental toxicants on sperm quality and potential risk for fertility in humans

**DOI:** 10.3389/fendo.2025.1545593

**Published:** 2025-05-21

**Authors:** Romualdo Sciorio, Pier Francesco Greco, Ermanno Greco, Luca Tramontano, Fathy M. Elshaer, Steven Fleming

**Affiliations:** ^1^ Fertility Medicine and Gynaecological Endocrinology Unit, Department Woman-Mother-Child, Lausanne University Hospital, Lausanne, Switzerland; ^2^ Villa Mafalda, Centre for Reproductive Medicine, Rome, Italy; ^3^ Department of Obstetrics and Gynecology, UniCamillus, International Medical University, Rome, Italy; ^4^ Département de Gynécologie-Obstétrique, Réseau Hospitalier Neuchâtelois, Neuchâtel, Switzerland; ^5^ Zoology Department, Faculty of Science, Al-Azhar university, Cairo, Egypt; ^6^ Discipline of Anatomy & Histology, School of Medical Sciences, University of Sydney, Sydney, NSW, Australia

**Keywords:** male infertility, sperm parameters, assisted reproductive technology, environmental pollution, oxidative stress

## Abstract

Semen quality is a key factor in male fertility, but defining normal reference values for semen parameters remain challenging. Over the past four decades, several authors have reported a noticeable decline in sperm parameters, raising concerns about male reproductive health. While the exact causes remain unclear, potential contributors include environmental pollution, endocrine disruptor chemicals (EDCs) and oxidative stress, with the latter becoming a growing concern. Environmental changes and increased exposure to EDCs, such as pesticides, herbicides, bisphenol A (BPA), phthalates, polychlorinated biphenyls (PCBs), and heavy metals, are believed to contribute significantly to the decline in sperm quality. These chemicals impact individuals from prenatal life through adulthood, potentially leading to long-term reproductive consequences. Overall, this review explores the relationship between environmental toxicants, including volatile organic compounds, EDCs, as well as oxidative stress and reduced male fertility. While a substantial body of research has found associations between these exposures and adverse fertility outcomes, some studies have reported no significant associations. The primary objective of this review is to provide a deeper understanding of the potential mechanisms between these environmental chemicals on testicular function and spermatogenesis. It also examines the broader evidence on the decline in sperm quality and explores its potential implications for overall fertility outcomes in humans. By doing so, the review will shed light on the broader public health implications of environmental pollutants and their impact on male reproductive health, emphasizing the need for further research in this critical area.

## Graphical Abstract

The figure illustrates the effect of environmental toxicants and plastics (macro and micro) on human reproduction and sperm quality. EDCs, Endocrine disrupting chemicals; PFAS, poly- and per-fluoroalkyl substances; BPA, bisphenol A; OCPs, organochlorinated pesticides; PCBs, polychlorinated biphenyls; ROS, reactive oxygen species.

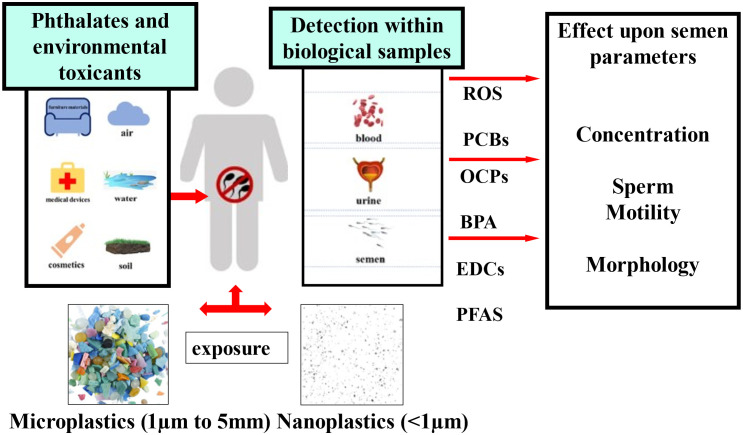

## Introduction: the global human sperm decline

Several published studies have recently highlighted a relevant decline in sperm count, nearly halved over the past few decades (1–6). Though the evidence for a decline in sperm parameters may be considered equivocal, male reproduction nevertheless seems to be at high risk. Various factors seem to contribute to this, including nutrition, environmental pollution, as well as an increase in cryptorchidism and testicular cancer, potentially linked to exposure to environmental estrogen-like endocrine disruptors (7, 8). Exposure to endocrine disruptors or high estrogen levels might cause a temporary reduction in testosterone, which has been reported by several authors, and may thereby impair male fertility (1, 3, 5). Notably, men generally do not normally seek medical attention for reproductive health unless they experience issues or difficulties in becoming parents. Interestingly, data have proposed a link between male subfertility and overall health status (9–15). A Danish study of 4,712 men found that semen analysis could serve as a biological marker for long-term morbidity and mortality, particularly related to cardiovascular alterations and diabetes mellitus (16). Additionally, men with low sperm number and motility were more likely to be hospitalized for several different pathologies and illnesses compared to those men with normal semen parameters. Among these hospitalized individuals, those with a sperm concentration of 195-200 million/ml were, on average, firstly admitted to hospital seven years later than counterparts with a sperm number less than 1 million/ml. The authors concluded that those results were independent of socioeconomic status and lifestyle factors, suggesting that normal sperm assessment could be associated with general health status in adult men (17). These findings were further supported by Capogrosso and co-workers (1).

## Environmental factors and sperm quality

Increased rates of infertility appear to coincide with rising global pollution. About 8–12% of couples worldwide experience infertility, with male factors being the predominant cause in up to 50% of cases (18). Male infertility affects about 7% of men worldwide (19). It is caused by a multitude of factors, including hormonal, genetic, behavioral, iatrogenic, environmental, and lifestyle variables, as well as congenital defects (20). Given that environmental toxins are widespread in today’s world, pollution has become a major factor contributing to the rising trend of male infertility (21, 22). The primary indicator of male fertility is semen quality (23). It has been observed that spermatogenesis, steroidogenesis, and sperm function are adversely affected by environmental pollution, which lowers male fertility and harms semen quality (24, 25). There is limited information regarding the direct effects of environmental chemicals on human spermatogenesis, even though chemicals found in industrial waste, pesticides, insecticides, herbicides, food additives, and other substances seem to adversely affect spermatogenesis in adult men. The available studies are mainly conducted in workplace settings, where individuals are exposed to these chemicals at high concentrations, rather than in the general population (26, 27).

### Air pollution

Air pollution has recently become a global concern, contributing to respiratory (28), cardiovascular (29), skin-related (30), and reproductive diseases (31, 32). Recent studies indicate that air pollution has a major effect on human fertility and sperm quality (21, 22, 33, 34). In India, ranked third for air pollution and with the second largest population (35), pollutants such as particulate matter, volatile organic compounds, ozone, nitrogen oxides, sulfur dioxide (SO_2_), carbon monoxide (CO), and radiation such as X-ray exposure, are major health threats (35, 36). Particulate matter, particularly PM10 (particles ≤ 10µm in diameter) is extremely dangerous, and enters the lungs and bloodstream after inhalation, leading to serious health issues (37). Finer particles, like PM2.5 (particles ≤ 2.5µm) present an even greater risk to health (34). Air pollution has been linked to increased sperm DNA fragmentation, sperm morphological alterations, and decreased sperm motility (38). A meta-analysis revealed a substantial negative correlation between air pollution levels and semen volume, sperm concentration, total sperm motility, morphology, and the DNA fragmentation index (22). A recent study on gaseous pollutants shows that exposure to SO_2_ considerably reduces sperm parameters across all exposure windows (39). Both SO_2_ and nitrogen dioxide (NO_2_) significantly affect sperm concentration and motility, especially during the early stages of spermatogenesis. A study by De Rosa and collaborators found that tollgate workers exposed to car exhaust had lower total sperm motility than nearby residents (40). Lead and nitrogen oxides from vehicle exhaust significantly impaired sperm quality. Calogero and co-authors reported that tollgate workers had high levels of sperm DNA fragmentation and damaged sperm chromatin compared to healthy, unexposed men (41). Ozone, a major air pollutant, is linked to defective sperm morphology, with increasing numbers of men reporting infertility due to abnormal sperm morphology (42). PM2.5, a primary cause of haze, has also been implicated in male infertility (22, 33, 34, 43). Studies show that sperm exposed to PM2.5 exhibit a higher frequency of morphological defects and cytoplasmic droplets (44). Additionally, sperm motility, concentration, total sperm count, sperm head shape, and overall semen quality are negatively correlated with PM2.5 exposure (45). Although the precise mechanisms by which air pollution causes male infertility are still unclear, several factors may help explain this link:

- Heavy Metals and PAHs: Car exhaust contains heavy metals like lead, zinc, and copper, as well as polycyclic aromatic hydrocarbons (PAHs), which have estrogenic, antiestrogenic, and antiandrogenic properties. These chemicals might impair gametogenesis and gonadal steroidogenesis, leading to infertility (46). PM2.5 accumulation in reproductive organs via placental and blood-testis barriers can also disrupt hormone levels and contribute to infertility (47).
- Oxidative Stress: Increased oxidative stress induced generation of reactive oxygen species (ROS), which results in lipid peroxidation, fragmentation of sperm DNA, and infertility (46).
- DNA Damage and Epigenetic Changes: Changes in gene expression and DNA methylation result in male infertility because of sperm DNA alteration brought on by the creation of DNA adducts, particularly with exposure to PAHs (46, 48).

## Endocrine-disruptor chemicals

Many chemical compounds commonly used in daily life have the potential to impact the vertebrate neuroendocrine system, which plays a crucial role in maintaining homeostasis and regulating essential processes such as development, growth, metabolism, and reproduction (49, 50). Over recent years, the release of various chemical pollutants, including pesticides, flame retardants, alkylphenols, polychlorinated biphenyls (PCBs), phthalates, and metals has significantly increased. Chemicals that mimic or interfere with the actions of naturally occurring hormones are classified as endocrine disrupting chemicals (EDCs) (51). These EDCs are defined as exogenous agents that disrupt the production, release, transport, metabolism, binding, action or elimination of natural hormones in the body. EDCs consist of a wide range of both natural and synthetic substances, most of which are released into natural waters due to anthropogenic activities. They enter living organisms through various routes, including air, soil, water and food, with the aquatic environment serving as the primary route of transmission. Once in the water, these substances can bioaccumulate through the food chain, which increases human exposure, particularly through the consumption of fish and seafood (52). Most of the time, environmental contaminants are typically transferred to humans unintentionally during daily activities, mainly absorbed through the skin, inhalation or ingestion (53, 54). Over 90% of the overall amount of chemical exposure occurs through dietary intake, which is the primary pathway for EDCs and other compounds to enter the human body (55). The adverse effects of these chemicals on the reproductive function of aquatic species is well documented, an example being the significant decline of fish populations in freshwater systems (56–59). Also, there is evidence suggesting that EDCs may be responsible for a skewed sex ratio at birth, with a higher incidence of male births being observed in some populations (60–64). Among this group of chemicals, steroidal estrogens (e.g. estrone, 17β-estradiol and 17α-ethinylestradiol) and phenolic xenoestrogens (e.g. alkylphenols and bisphenol A) are of particular concern (65). The growing concern over environmental chemicals is largely due to their association with various human health disorders, including testicular cancer, falling sperm counts, endometriosis, precocious puberty, and breast cancer (66). It is well-established that organisms have evolved sensitivity to both endogenous and exogenous chemical signals, allowing them to adapt to physical, chemical or biological stimuli while maintaining internal homeostasis. However, this sensitivity to environmental cues also makes organisms vulnerable to inadvertent and potentially harmful chemical signals from the surrounding environment (67). The long-term exposure to EDCs raises critical concerns about the risks to human health. As these chemicals accumulate in the environment, the risk to both wildlife and human populations becomes more evident, especially related to reproductive function. As such, the growing body of evidence underscores the need for increased regulation of these chemicals, particularly those that are known to exhibit endocrine-disrupting properties (51, 65, 68–71). Additionally, certain EDCs, also known as “obesogens,” have been implicated in the promotion of obesity, insulin resistance, and increased risk of type II diabetes (72–74). These metabolic disorders, in turn, seem to be significant risk factors for cardiovascular disease (75, 76). The effects of EDCs also extend to bone metabolism. Some persistent organic pollutants (POPs) have been shown to alter the processes involved in bone development and turnover, likely through their estrogenic and anti-estrogenic actions (77, 78). Additionally, numerous EDCs have been shown to either depress the immune system or cause hyper-immunity, leading to altered immune responses to infections and an increased risk of cancer (72, 79). Although it is still up for debate whether the effects of EDCs on the immune system qualify as “endocrine” effects, there is no denying that they pose a serious risk to human health. The widespread presence of these chemicals in the environment and their ability to interfere with critical hormonal functions pose a significant threat to public health. Understanding the mechanisms by which EDCs impair reproductive function is essential for developing effective strategies to mitigate their impact on both wildlife and human populations. As research continues to reveal the full extent of these chemicals’ effects, public awareness and policy action will be key in reducing exposure and minimizing health risks associated with EDCs (Green^51^, ^70^, ^71^).

### Effect of endocrine disruptors on sperm quality

**Figure 1:**
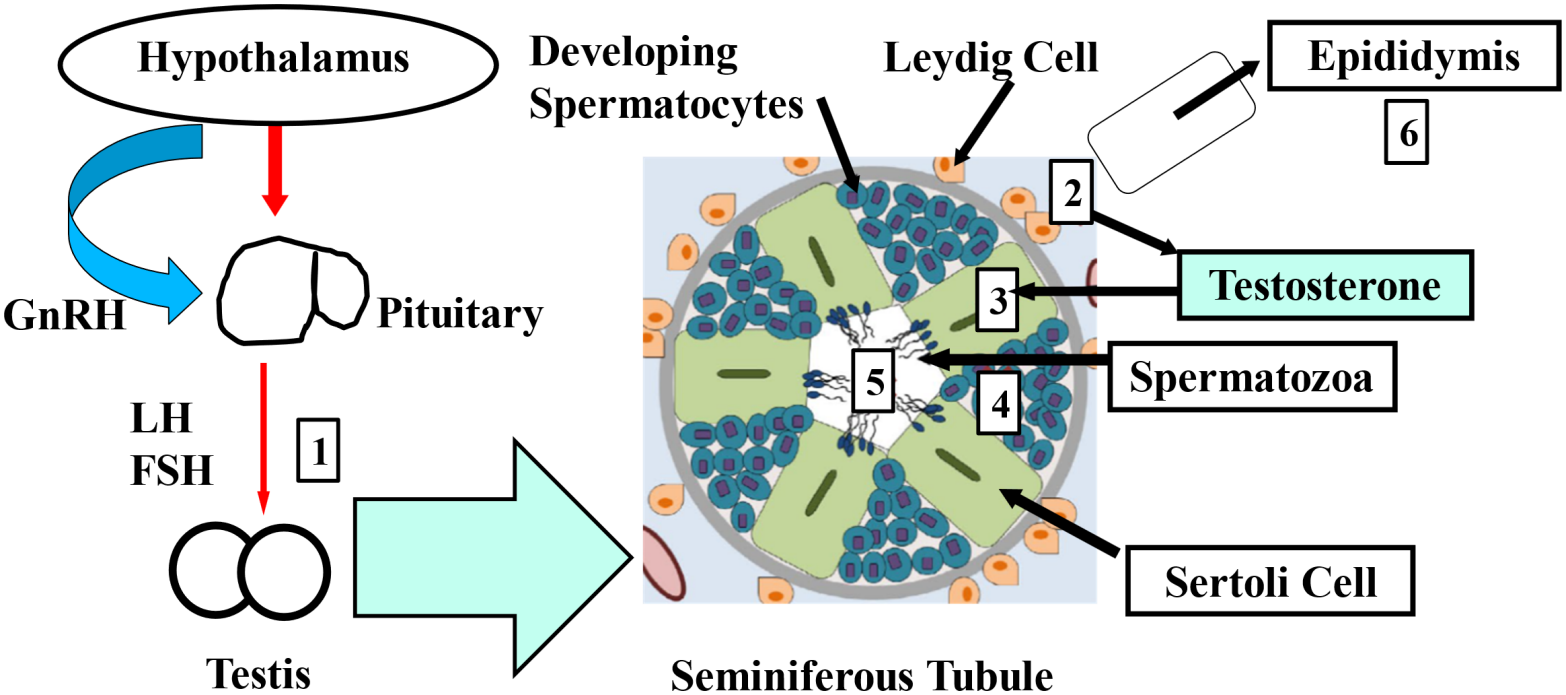
The primary mechanisms through which EDCs affect sperm quality are outlined as follows 1-6. GnRH: gonadotropin-releasing hormone, LH: luteinizing hormone, FSH: follicle-stimulating hormone. (1) Interference with testicular gonadotropin receptors, (2) disruption of Leydig cell steroidogenesis, (3) damage to Sertoli cells, (4) inhibition of spermatocyte development, (5) disruption of mature sperm, (6) alteration of epididymal sperm modification.

Several studies have provided substantial evidence that EDCs can mimic or block steroid hormones by acting as their agonists or antagonists, disturbing normal hormone-regulated processes, particularly those related to sexual development and reproduction (80–82). EDCs, various compounds capable of disturbing the endocrine system in both wildlife and humans, have raised significant concern among the public and toxicologists (68, 69, 83, 84). Environmental pollutants, such as organochlorinated pesticides (OCPs) and PCBs have been linked to “endocrine disruptor” effects (85, 86). These POPs harm human health in several ways, causing birth defects and posing neurotoxic, hepatotoxic, nephrotoxic, immunotoxic, and carcinogenic consequences (72, 87). When the term “endocrine disruption” was first introduced in 1991, research mainly focused on the estrogenic effects of these chemicals, leading to their initial classification as xenoestrogens (88, 89). Various *in-vivo* and *in-vitro* studies have reported the presence of many substances with estrogenic, anti-estrogenic, androgenic, and anti-androgenic properties (90, 91). The adverse reproductive effects of EDCs are well-documented, as they interfere with endocrine function by blocking receptor activity. These regulatory processes are crucial and closely linked to sperm production (14, 15, 51, 70, 71, 92–94). Moreover, sperm production and quality are regulated at multiple levels. The hypothalamus releases gonadotropin-releasing hormone (GnRH), which in turn stimulates the anterior pituitary gland to release luteinizing hormone (LH) and follicle-stimulating hormone (FSH). Disruption at any stage of this process can lead to damage in sperm quality. **Figure 1** illustrates the main points where EDCs exsert their influence. Testicular damage may involve increased spermatocyte apoptosis due to Sertoli cell dysfunction or the overexpression of apoptotic proteins (95, 96). Sertoli cells nourish developing spermatocytes, removing excess cytoplasm and promoting testosterone-driven spermatogenesis. When Leydig cells fail to produce testosterone, androgen receptor-mediated gene transcription necessary for spermatogenesis might be impaired. Some research indicates that EDCs such as BPA may inhibit ATP production (97), potentially by disrupting mitochondrial function, which could reduce sperm motility. Additionally, an altered hormonal environment caused by EDCs might contribute to aneuploidy in sperm and potential transgenerational effects. However, many of these proposed mechanisms require further validation through clinical studies to better understand how EDCs affect male infertility (97, 98). As a result, EDCs pose significant risks to both human and environmental health, particularly concerning reproductive function. Their ability to interfere with natural hormone function emphasizes the need for further research and stronger regulations. As evidence of their harmful effects increases, it is critical to prioritize public health policies aimed at reducing exposure and mitigating the long-term consequences of endocrine disruption on both wildlife and human populations.

### Pollution by plastics and plasticizers

Using machine learning and probabilistic material flow analysis, it has been estimated that the world produces 52 million metric tons of macroplastics per year, with significant levels of plastic pollution accumulating within our environment (99). The production of macroplastics continues unabated, despite the dire warning of their threat to reproductive health (100). Plasticizers having an approximate half-life of six hours, such as BPA, do not bioaccumulate in the body (101, 102) and are excreted through urine. Plasticizers are commonly found in populations; in the US, 92.6% of individuals aged 6 and older have BPA present in their body (103). Even though plasticizers are not retained in adipose tissue, everyday exposure to these compounds raises questions about their potential to affect hormones (102). Humans may also inhale certain environmental pollutants that have volatilized and become contaminated (24, 104). Inhalation can be a significant mode of exposure, particularly for volatile and semi-volatile substances (105). Apex predators, such as polar bears, are frequently found to contain environmental contaminants. It is believed that the presence of EDCs in the tissues of animals living in remote locations indicates that these chemicals are distributed over great distances via both air and ocean currents (24, 106). An essential habitat for possible exposure to airborne particles and chemicals is the indoor living or working space. Another important way that workers in hazardous environments might become contaminated is through occupational exposure to EDCs (105). One of the primary EDCs, extensively used in the production of epoxy resins, hard polycarbonate plastics, and many other materials, is BPA. By binding competitively to many kinds of estrogen receptors, BPA imitates the effects of estrogen (107). It has also been found that BPA decreased serum levels of thyroid hormone, hormone production, and the release of hypothalamic steroid hormones (108), lowered levels of male gonadotropin hormone (109), and caused abnormal embryonic development and impaired implantation (110). In a similar vein, chronic BPA exposure has been shown to affect ovarian reserve in non-pregnant mice (111) and disrupt the estrous cycle (112). Additionally, a different investigation found that low concentrations of BPA induced oxidative stress in the testis *in-vitro* (96). Due to public health concerns about the toxic effects of BPA, its application is restricted especially in the US and is replaced by “BPA-Free” products that contain substitutes such as bisphenol-F, bisphenol-B and bisphenol-S (BPS) (113).

## Microplastics, nanoplastics and sperm quality

Microplastics (1µm to 5mm in diameter) and nanoplastics (<1µm in diameter) can either be manufactured as such (primary) or form as a result of the breakdown of larger plastics (secondary), defined by both their size and chemical composition. When exposed to natural environmental forces, such as mechanical friction, extreme heat, and ultraviolet radiation, plastics undergo physical and chemical aging, breaking down into smaller particles, typically within the nanometer to micrometer range in diameter. These smaller particles can then be widely distributed across the environment, appearing in the atmosphere, soil, oceans, and even in the food and water we consume (6). Additionally, microplastics can adsorb and release POPs and toxic heavy metals, facilitating their transport and potential bioaccumulation in the environment. Polyethylene (PE), polypropylene (PP) and polystyrene (PS) are the most common microplastic polymers found in the marine environment. Studies have confirmed the presence of microplastics in human feces (114) and urine (115), demonstrating that they can be ingested, are small enough to cross cell membranes, and can be excreted. In a study involving Italian volunteers Raman microspectroscopy identified several types of microplastics in urine, including polyethylene vinyl acetate (PVA), polyvinyl chloride (PVC), PP, and PE (115). Given our increasing exposure to microplastics in daily life, there is a growing concern regarding their potential negative impacts on reproductive health and male fertility (116). With respect to neuroendocrine control of male reproduction, a significant inverse correlation between the dosage and duration of exposure to PS microplastics and serum levels of FSH, LH and testosterone, has been observed in male rats and mice (117–119). After just 24 hours exposure to environmental levels (100µg/L and 1mg/L) of PS microplastics within the drinking water, PS microparticles accumulated within the testis, with chronic exposure leading to testicular inflammation, disruption of the blood-testis barrier (BTB), and a decline in testosterone serum levels (117). Furthermore, sperm morphology, DNA integrity and viability were also impaired. *In-vitro* studies using primary cultures of mouse Leydig cells showed that PS microplastics adhered to and were internalized by these cells, causing downregulation of the LH receptor, steroidogenic acute regulatory protein (StAR), and steroidogenic enzymes, resulting in a decrease in testosterone production (119). The BTB is essential for maintaining male reproductive function and is generally considered impermeable to most toxicants (120). However, nanoplastics in particular, have been shown to accumulate within Sertoli cells (121). Interestingly, several studies have demonstrated that PS microplastics reduce the expression of various proteins critical to BTB integrity, including basal ectoplasmic specialization protein, β-catenin, claudin-11, connexin-43, N-cadherin, occludin, and zona occludens-1 (122, 123). Moreover, PS microplastics induce oxidative stress, damage seminiferous tubules, and cause apoptosis in spermatogenic cells, which results in reduced sperm concentration and motility, as well as increased abnormal sperm morphology (122). One potential mechanism by which microplastics compromise BTB integrity is through the suppression of the mammalian target of rapamycin (mTOR)/protein kinase B (also known as Akt) pathway via their generation of ROS. In this respect, mTOR and focal adhesion kinase (FAK) regulate F-actin organization within the cytoskeleton of the BTB (124), and PS microplastics have been shown to disrupt this regulation via generation of ROS (123). PS microplastics have also been detected in the epididymis of all bulls tested, with a mean concentration of 0.37µg/mL (125). Furthermore, *in-vitro* exposure to comparable concentrations of PS reduced bovine sperm motility and impaired blastocyst development, with evidence of increased formation of ROS and apoptosis (125). Using the mouse model, several studies have demonstrated that both PS micro- and nanoplastics can disrupt perinatal testicular development, reduce fertility, and even cause infertility in the male (126–128). In a more recent study, it was demonstrated that daily oral ingestion of PS microplastics (1mg/dL or 3mg/dL) for 28 or 56 days resulted in their detection within the testis (129). After 56 days of exposure to either concentration of PS, there was a significant decrease in sperm count and motility, along with a marked increase in sperm morphological abnormalities. Clinical studies on the impact of microplastics on male factor infertility are scarce. However, a multi-site study conducted in China examined the association between mixed exposure to microplastics and dysfunction of spermatogenesis (130). Semen and urine samples were collected from 113 participants across three regions. Using Raman microscopy, microplastics were detected in all semen and urine samples, with the highest detection rates for PS, PE and PP. Interestingly, polytetrafluoroethylene (PTFE) exposure was significantly associated with decreased semen quality, including decreased sperm count and concentration. Additionally, multi-linear regression analysis showed that exposure to each additional polymer type correlated with a significant decrease in total sperm count, concentration and progressive motility (130). Another recent study used advanced sensitive pyrolysis-gas chromatography/mass spectrometry to quantify 12 types of microplastics within the testis of the human and the dog (131). Microplastics were found to be present within all testis samples, with significant inter-individual variability. The mean concentration of microplastics was 122.63 µg/g in the dog testis and 328.44µg/g in the human testis. Interestingly, a negative correlation was observed between the presence of specific polymers such as PVC and polyethylene terephthalate (PET) and the normalized weight of the testis (131). Of even greater concern, nanoplastics are likely to be more pervasive than microplastics due to their smaller size and larger surface area-to-volume ratio, which enhances their ability to adsorb and release EDCs and toxic heavy metals. Consequently, further research is urgently required to determine whether different particle sizes and different polymers have differential impacts on male and female fertility.

## Perfluoroalkyl and polyfluoroakyl substances and sperm assessment

One diverse group of POPs, known as poly- and per-fluoroalkyl substances (PFAS), is represented by thousands of synthetic perfluorinated organic chemicals (PFCs) typically used in the manufacture of non-stick cookware and food packaging. Though some more persistent longer carbon chain PFAS, such as perfluorooctanoate (PFOA) and perfluorooctane sulfonate (PFOS), known as “forever chemicals”, have been phased out in manufacturing since the turn of the century, their past usage on a grand scale has resulted in their bioaccumulation and ubiquitous persistence within the environment. Alarmingly, epidemiological evidence has long associated exposure to PFAS with testicular dysgenesis, including testicular cancer and impaired semen quality (132). Though a later systematic review of their impact on human fertility proved equivocal, in the male at least (133), a more recent meta-analysis has revealed that concentrations of PFOA and perfluoronanoic acid (PFNA) are inversely associated with sperm progressive motility (134). Furthermore, exposure to PFOA *in utero*, measured in maternal blood samples from week 30 of pregnancy, has been associated with higher levels of gonadotrophins (FSH and LH) in the systemic circulation, and reduced sperm count and concentration in 169 adult male offspring (135). A similar investigation of 864 young men from the Fetal Programming of Semen Quality (FEPOS) cohort was conducted recently (136). First trimester plasma samples from their mothers were retrieved from the Danish National Biobank and were analyzed for the presence of up to 15 PFAS. Using weighted quantile sum regression and negative binomial regression, combined maternal exposure to PFAS was associated with lower sperm concentration, count, and higher non-progressive sperm motility and immotility in their offspring (136). Therefore, coincident with the global decline in male fertility, the enduring presence of PFAS should be of great concern to reproductive health specialists (137). Epidemiological studies are, by nature, plagued by multiple confounding factors, making it difficult to assign causality but, nevertheless, provide large data sets for examining possible associations between PFAS and sperm quality. One such study, investigated the possible association between the serum levels of 10 different perfluoroalkyl acids (PFAAs) and testicular function in 105 men from the general population (138). Using liquid chromatography-tandem mass spectrometry with electrospray ionization, it was found that men with high combined levels of PFOA and PFOS had a significantly lower median total normal sperm count of 6.2 million in their ejaculate versus 15.5 million in men with a low combined level of PFOA and PFOS (138). A multi-geographical study investigated PFAS and their possible association with reproductive hormones and sperm quality in 604 partners of pregnant women (139). There was a slight increase in sex hormone-binding globulin (SHBG) and *in situ* terminal deoxynucleotidyl transferase dUTP nick-end labelling (TUNEL), suggestive of reduced bioavailability of testicular steroids and increased sperm DNA fragmentation. However, no consistent evidence was found for a significant correlation between exposure to PFAS and sperm DNA fragmentation, apoptosis, or reproductive hormones (139). However, a later study did find a significant negative association between exposure to PFCs and sperm quality (140). Contamination with PFCs was observed within the whole blood of 58% of subjects and this was associated with a significant increase in alteration of semen parameters compared to those in whom PFCs were not detected. Furthermore, using fluorescent *in situ* hybridization (FISH) for chromosomes 18, X and Y, and TUNEL coupled to flow cytometry for sperm DNA fragmentation, sperm disomy and diploidy rates, and the DNA fragmentation index were significantly increased in PFC-positive versus PFC-negative individuals (140). Another study included the male partners of 501 couples planning pregnancy (141). Men had blood collected and provided a baseline semen sample plus another approximately one month later. Using tandem mass spectrometry, seven PFCs (perfluorosulfonates, perfluorocarboxylates, and perfluorosulfonamides) were quantified within the serum. After adjusting for confounders and modelling repeated semen samples, linear regression analysis showed that perfluorooctane sulfonamide (PFOSA) was associated with smaller sperm heads, lower DNA stainability and higher bicephalic and immature spermatozoa (141). A study specifically focused on the Pearl River delta, a region in China labelled one of the “world factories,” investigated PFAAs within the blood and semen of 103 participants (60). These men were found to have higher levels of PFAAs than men in other regions within China. Also, there was a significant inverse correlation between the levels of perfluoro-n-pentanoic acid (PFPeA), perfluorohexanoic acid (PFHxA), perfluorobutanoic acid (PFBA), perfluorobutanesulfonic acid (PFBS), perfluorohexanesulfonic acid (PFHS), PFOA, and PFOS with sperm motility (60). In a broader study, matched semen and serum samples were collected from 664 men from a cross-sectional population of couples undergoing their first assessment of fertility (142). Using mass spectrometry, 16 target PFAS were analyzed and their association with semen quality parameters was evaluated by multivariable linear regression analysis. Seminal PFOA, PFOS and emerging chlorinated polyfluorinated ether sulfonate (6:2 Cl-PFESA) were significantly associated with a lower percentage of progressively motile spermatozoa and a higher percentage of sperm DNA fragmentation (142). The mechanism by which PFAS impair sperm quality is largely unknown. However, a recent *in-vitro* study using exposure of spermatozoa to environmentally relevant concentrations of a cocktail of PFAS has attempted to address this in the mouse model (143). Interestingly, a three-hour exposure to PFAS *in-vitro* did not affect the sperm functional profile, in terms of capacitation or fertilization rates, but did significantly delay the developmental progression of *in-vitro* fertilized day 4 preimplantation embryos, which suggested an alternative stress-mediated impact at fertilization. Clearly, further research is warranted to identify the mechanisms and threats that PFAS present to male fertility and human health.

## Oxidative stress and sperm quality

**Figure 2:**
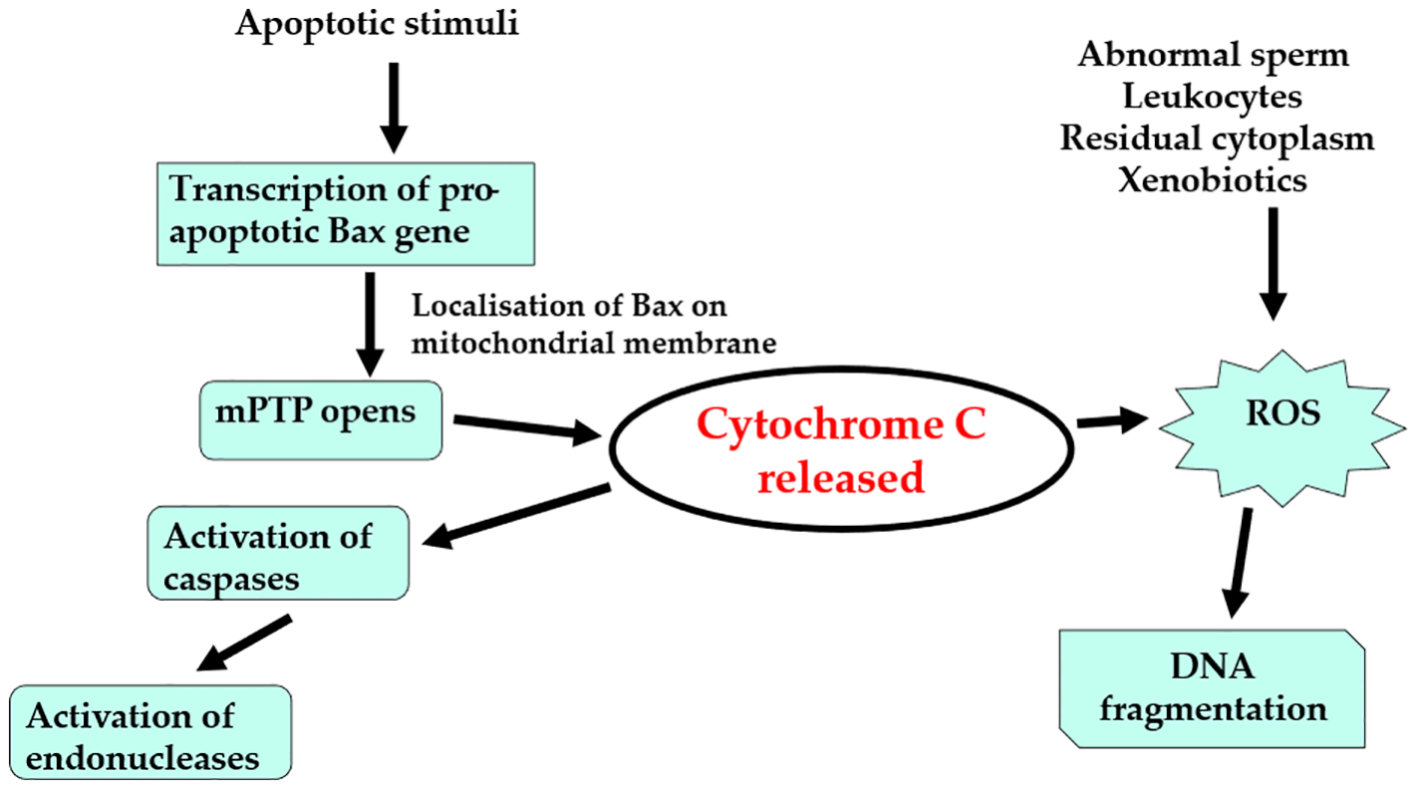
Concurrent pathways involved in ROS, and sperm DNA damage/fragmentation. Adapted from (144). ROS: reactive oxygen species; DNA: deoxyribonucleic acid; mPTP: mitochondrial permeability transition pore.

Recent studies have highlighted that one of the major causes of testicular damage is oxidative stress, which leads to an increase in the production of ROS (**Figure 2**). This imbalance can result in significant changes in protein production and DNA damage in testicular cells (144, 145). The production of ROS is a condition in which the natural equilibrium between oxidants and antioxidants is disrupted, causing the generation of free radicals. These free radicals contain an uneven number of electrons, making them highly reactive with other chemical compounds or molecules. This reactivity triggers a cascade of chemical reactions than can be toxic to cells and tissues, including gametes and embryos (146–149). Increased ROS at the level of the testis have also been associated with dysfunction in site-specific hypermethylation. This occurs either through the upregulation of DNA methyltransferases (DNMTs) or the formation of new complexes involving these enzymes (148, 150, 151). It is important to emphasize that sperm epigenetic changes due to oxidative stress may be secondary to additional factors, including sperm manipulation during ART treatment, or individual patient characteristics, such as age, health conditions, or lifestyle (150, 152, 153). Nevertheless, research indicates that oligozoospermic men tend to experience more pronounced epigenetic changes compared to men having normal sperm parameters (154–156). Moreover, an increase in seminal ROS production and a corresponding decrease in antioxidant enzyme activity have been linked to a variety of sperm alterations, including chromosomal abnormalities, micronuclei formation, changes in sperm membrane potential, as well as an increased rate of apoptosis and DNA fragmentation (148, 149, 151, 157–159). Sperm DNA damage caused by ROS has serious implications for embryogenesis, potentially leading to increased risks of implantation failure or miscarriage. The extent of sperm DNA damage, whether through single or double-strand breaks, can influence the ability of the oocyte to repair the damage at fertilization or post fertilization, impacting the overall chances of a successful pregnancy (160). The primary targets of ROS-induced damage in the testis include Leydig cells, seminiferous tubules, and spermatozoa. ROS disrupt the normal function of Leydig cells, leading to decreased testicular steroidogenesis and reduced testosterone, which in turn affects spermatogenesis and can lead to infertility (149, 157, 158, 160). In a study by Desai and collaborators (161), it was shown that increased ROS levels can induce sperm DNA damage and even sperm death. Another study found a strong association between oxidative stress, increased lipid peroxidation, and dysfunction in the body’s antioxidant defense functions. The sperm plasmalemma is particularly vulnerable to lipid peroxidation due to its high content of polyunsaturated fatty acid. The byproducts of lipid peroxidation can damage the sperm plasmalemma, disrupt the function of mitochondrial proteins involved in the electron transport chain, and ultimately impair sperm motility and fertilization potential (162). It is well established that sperm motility, acrosome reaction, and binding to the oocyte are highly sensitive to oxidative stress. ROS can induce a loss of membrane fluidity and compromise the integrity of the sperm membrane, both of which are crucial for successful fertilization (163). This loss of membrane integrity can significantly impair sperm function, reducing motility, compromising the acrosome reaction, and hindering the sperm’s ability to bind effectively with the oocyte during fertilization. As a result, oxidative stress plays a critical role in both male fertility and reproductive outcomes, influencing sperm quality and the ability to conceive. Interestingly, recent research has focused on the potential for antioxidants to mitigate the effects of ROS on sperm and overall testicular health. Antioxidant supplementation, such as with vitamins C and E, has been shown to reduce oxidative stress in sperm, potentially improving sperm motility and DNA integrity. However, the effectiveness of such interventions remains debated, and more research is needed to understand the optimal dosages and specific antioxidants that may benefit male fertility (164, 165). Moreover, the balance between oxidative stress and antioxidant defense mechanisms is highly dynamic, and what might be beneficial in one context may not be effective in another. Ultimately, the growing body of evidence underscores the importance of oxidative stress as a major factor influencing male fertility. Both environmental and lifestyle factors can increase ROS production, leading to sperm DNA damage and compromised fertility. The complex interplay between oxidative stress, epigenetic modifications, and sperm function highlights the need for further research into the mechanisms underlying male infertility. By understanding these processes more thoroughly, strategies can be developed to mitigate oxidative stress and its impact on sperm quality, providing hope for improved fertility treatments and outcomes for men struggling with infertility.

## Oxidative stress and mitochondrial function

Mitochondrial function is vital for reproductive health, as mitochondria provide the energy needed for sperm motility, essential for navigating the female reproductive tract and fertilizing the oocyte. Disruptions in mitochondrial metabolism, particularly within the electron transport chain, can be caused by an excessive production of ROS (166, 167). Increased mitochondrial ROS production is a key factor in sperm DNA fragmentation, reducing sperm viability and fertilization potential (168). DNA damage is a significant cause of male infertility, as it affects embryo development and increases the risk of miscarriage. The impact of mitochondrial dysfunction extends beyond DNA damage and motility issues. ROS can also disrupt important sperm functions by overwhelming antioxidant defense systems. Seminal enzymes like superoxide dismutase (SOD), catalase (CAT), and glutathione peroxidase (GPx) protect sperm by neutralizing ROS. These antioxidants help maintain a balance between ROS production and clearance. However, when mitochondrial ROS production exceeds the capacity of these protective enzymes, sperm cells become vulnerable to oxidative damage (163, 169). Damaged sperm membranes further impair motility, survival, and the acrosome reaction, a crucial process for fertilization. If the sperm cannot undergo the acrosome reaction, it cannot penetrate the egg’s outer layers, making fertilization impossible. In ART, where sperm quality is critical, oxidative stress poses a significant challenge, and sperm impairment might lead to a decrease in fertilization rate and poor embryo development. Managing ROS levels is crucial in ART: and usage of antioxidants can protect sperm from oxidative damage by neutralizing excess ROS and supporting mitochondrial function. Supplementation with antioxidants such as vitamins C and E, coenzyme Q10, and N-acetylcysteine has been explored as a way to reduce oxidative stress and improve sperm quality in men undergoing fertility treatments (164, 170–172). By restoring the balance between ROS production and antioxidant defense, these strategies may enhance sperm quality and increase the chances of successful fertilization. However, the clinical use of antioxidants is still under investigation. Optimal dosages, specific antioxidants, and timing of supplementation require further research to understand how best to support mitochondrial function and sperm quality in assisted reproduction. Finally, mitochondrial function is critical for sperm health: excessive ROS production damages sperm DNA, impairs motility, and compromises membrane integrity, negatively impacting fertility. Understanding and managing the balance between ROS and antioxidants is essential for improving outcomes in ART. By addressing oxidative stress with targeted antioxidant therapies, it may be possible to mitigate ROS effects on sperm function, ultimately enhancing fertility treatments for couples struggling with infertility.

## Conclusive remarks

This manuscript addresses the growing concern that environmental pollution negatively affects sperm quality, contributing to the decline in male reproductive health. Environmental toxins, such as heavy metals, pesticides, industrial chemicals, EDCs, microplastics, and oxidative stress, may impair hormonal balance, sperm production, and fertility. Over time, these toxins accumulate in the body, leading to reduced sperm count, motility, and DNA fragmentation, which significantly affect fertility (164). These toxins are commonly found in contaminated air, water, and food, posing a persistent risk to male reproductive health. Air pollution is another major factor influencing male infertility. Prolonged exposure to pollutants like particulate matter, NO_2_, and CO can harm sperm quality. These pollutants generate ROS, which might damage sperm DNA, membranes, and mitochondria. Oxidative stress reduces sperm motility, viability, and the likelihood of successful fertilization (163, 173). With air pollution widespread in urban areas, its impact on male fertility must be further investigated. EDCs, found in pesticides, plastics, and personal care products, disrupt hormonal systems involved in reproduction. These chemicals mimic or block hormones like estrogen and testosterone, which are critical for sperm production and function. Phthalates, BPA, and other plasticizers have been linked to reduced sperm count, motility, and testosterone levels (174, 175). Given the ubiquity of EDCs in everyday products, avoiding exposure is challenging, and their long-term effects on male fertility are still under investigation. Microplastics, present in nearly every ecosystem, pose a growing threat to male reproductive health. Their small size allows them to be ingested by humans and animals through food and water. Recent studies show that microplastics can accumulate in human tissues, including the testis, potentially contributing to oxidative stress and disrupting sperm function. Oxidative stress is a central mechanism through which many environmental factors, such as toxins, air pollution, EDCs, and microplastics, contribute to male infertility. An imbalance between ROS production and antioxidant defenses damages sperm DNA, impairs motility, and reduces fertilization potential. Increased exposure to environmental pollutants exacerbates oxidative stress, further compromising sperm quality. In response to these concerns, scientists and public health experts urge governments to prioritize male reproductive health by increasing research funding and implementing policies to reduce harmful environmental exposures. Large-scale studies are necessary to establish definitive links between environmental factors and male infertility. Governments must also regulate substances like industrial chemicals, air pollution, and plastics to protect male reproductive health. Urgent action is needed to mitigate these environmental hazards and safeguard reproductive health for future generations.
